# Supplementary material for: Splice-Junction-Based Mapping of Alternative Isoforms in the Human Proteome
Source: Cell Rep. Author manuscript; Available in PMC 2020 Jan 15. (PMC6961840; doi:10.1016/j.celrep.2019.11.026)
Supplement: 2 [file NIHMS1546469-supplement-2.zip › DF1/PXD006675/VentricularSeptum/VentricularSeptum_29_LDB3_VVVNSPAK.pdf]

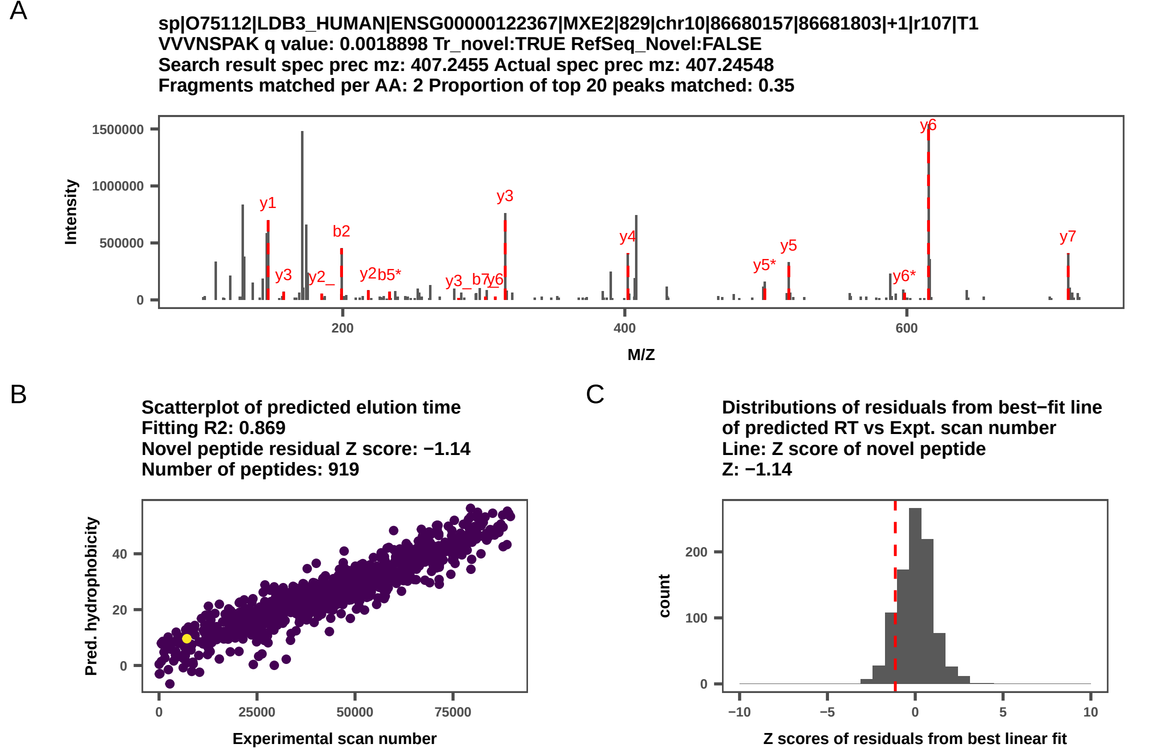
A
sp|O75112|LDB3_HUMAN|ENSG00000122367|MXE2|829|chr10|86680157|86681803|+1|r107|T1
VVVNSPAK q value: 0.0018898 Tr_novel:TRUE RefSeq_Novel:FALSE
Search result spec prec mz: 407.2455 Actual spec prec mz: 407.24548
Fragments matched per AA: 2 Proportion of top 20 peaks matched: 0.35
Intensity
1500000
1000000
500000
0
y1
y3
y2_
b2
y2
b5*
y3
y3_
b7
y6
y4
y5*
y5
y6
y6*
y7
200
400
600
M/Z
B
Scatterplot of predicted elution time
Fitting R2: 0.869
Novel peptide residual Z score: −1.14
Number of peptides: 919
Pred. hydrophobicity
40
20
0
0
25000
50000
75000
Experimental scan number
C
Distributions of residuals from best−fit line
of predicted RT vs Expt. scan number
Line: Z score of novel peptide
Z: −1.14
count
200
100
0
−10
−5
0
5
10
Z scores of residuals from best linear fit
